# Knockout of PARP2 in T cells leads to spontaneous colitis with distinct segmental characteristics in PARP1-deficient mice

**DOI:** 10.3389/fimmu.2026.1741798

**Published:** 2026-05-19

**Authors:** Haoran Ke, Dongwoog Kim, Máté Bencsics, Bálint Bányai, Roland Csépányi-Kömi, Péter Sasvári, Françoise Dantzer, Najat Hanini, José Yélamos, Anna-Mária Tőkés, Ágnes Korsós-Novák, Rita Benkő, Eszter M. Horváth

**Affiliations:** 1 Department of Physiology, Semmelweis University, Budapest, Hungary; 2 UMR7242, Biotechnology and Cell Signaling, Centre National de la Recherche Scientifique (CNRS)/Université de Strasbourg, Strasbourg, France; 3 Hospital del Mar Medical Research Institute (IMIM), Barcelona, Spain; 4 Department of Pathology, Forensic and Insurance Medicine, Semmelweis University, Budapest, Hungary; 5 Department of Pathology, Toldy Ferenc Hospital and Outpatient Clinic of Cegléd, Cegléd, Hungary

**Keywords:** colitis, IBD, PARP1, PARP2, T cells

## Abstract

**Background and aim:**

Suppression of poly(ADP-ribose) polymerase (PARP) activity, either PARP1 or PARP2, was proven to be beneficial in experimental colitis. The absence of PARP2 exclusively in T cells (T-PARP2-KO) was also shown recently to exert similar protection. Nevertheless, lower PARP1 expression in the intestines and reduced PARP activity in mononuclear cells from IBD patients were reported. Thus, we would like to investigate the effect of PARP2 deficiency in T cells superimposed on a PARP1 deletion background in the large intestines.

**Methods:**

DKO animals were generated from crossbreeding mice bearing single knockouts. The large intestines were examined by histopathology and various immune-based methods (immunohistochemistry, ELISA, and Western blot) to characterize the intestinal alterations through comparison with control and single knockout animals.

**Results:**

The DKO animals developed spontaneous intestinal inflammation in both the proximal and distal large intestines, reflected by both histological features and altered levels of the pro-inflammatory cytokine TNFα. In DKO animals, higher density of T cells and increased oxidative–nitrative stress were observed in the intestinal epithelium. Altered expression and activation of intracellular inflammatory signaling transduction proteins were found exclusively in the proximal segments, while the distal large intestines displayed elongated crypts despite the higher activation of caspase-3. The higher ratio of Ki-67-positive cells imply that the increased renewal of enterocytes is attributable to mucosal hyperplasia.

**Conclusions:**

PARPs may have essential roles in maintaining the integrity of large intestines, which are closely associated with lymphatic tissues. The combined genetic modification could potentially induce harmful alterations of the mucosa and the associated lymphatic tissue, leading to the occurrence of spontaneous inflammation with certain distinct segmental features.

## 1 Introduction

Inflammatory bowel disease (IBD) is a collection of diseases characterized by auto-inflammation in the alimentary tract, which largely hampers the life quality of patients and imposes great socio-economic burden (1). The gastrointestinal tract is constantly exposed to countless antigens from dietary or microorganism origins. The tightly regulated balance between responsiveness and tolerance from the mucosal immune system against these antigens precedes the normal physiology, while the disruption of the balance could result in structural or functional disturbance and pathologies (2). The pathogenesis of IBD is believed to be partially attributed to dysregulated members of the adaptive immunity, especially misbalance between certain subpopulation of T cells, e.g., helper T 17 cells (Th17) and regulatory T cells (Treg) (3).

Poly(ADP-ribose) polymerase (PARP) is a superfamily of eukaryotic nuclear enzymes which covalently modify proteins like transcription factors via ADP-ribosylation. PARPs participate in various processes of cellular metabolism, especially maintaining the genomic integrity (4). In recent years, the immunological roles of PARPs are also gradually explored. PARP1, the most abundant member of the PAPR family, was proven to promote inflammation in multiple diseases and conditions, e.g., rheumatoid arthritis, septic shock, IBD, while the suppression of PARP1 activity (e.g., pharmacological inhibitors such as PJ-34, nicotinamide, *Parp1^-/-^* genotype) could mitigate the inflammatory injuries (5). Nevertheless, the activity of PARP1 does not always exhibit a positive correlation with the extent of inflammation, and studies involving animal models of chronic autoimmune or auto-inflammatory diseases suggested a rather complex involvement of PARP1. The genetic deficiency of PARP1 was shown to enhance the inflammatory responses in both experimental autoimmune encephalitis mimicking multiple sclerosis and imiquimod-induced model of psoriasis (6, 7). Despite the proven benefits from PARP1 absence in experimental colitis, clinical studies have yet revealed facts that implicated the complex role of PARP1, e.g., the presence of autoantibodies against PARP1 in the sera of IBD patients, along with the increased transcriptional level but decreased translational level of PARP1 in the intestines of patients with active diseases (8–11). Another PARP homolog, PARP2, shares similar localization and functions as PARP1 (4). To a certain extent, PARP2 inhibition is also proven to improve inflammatory responses (12, 13). Distinctly, PARP2 has a decisive role in the development of T cells, while the deficiency of which could cause a higher p53-dependent apoptosis of double-positive thymocytes and impaired TCRα expression (14). On this basis, our working group demonstrated that the absence of PARP2 in T cells (T-PARP2-KO: *Cd4-Cre*, *Parp1^+/+^*, and *Parp2^f/f^*) ameliorates acute LPS-induced colitis (15).

Although PARP1 appeared to be waivable for T cell homeostasis, Navarro J. et al. and Moreno-lama L. et al. have discovered that T cells with double deficiency of PARP1 and PARP2 are more vulnerable to apoptosis, and the T-cell-mediated immune responses against certain pathogens or malignant cells are largely compromised (16, 17). Since the true double deficiency leads to death *in utero* (18), the above-mentioned studies were conducted in mice with global PARP1 deficiency (*Cd4-Cre*, *Parp1^-/-^*, and *Parp2^+/+^*) and T cell PARP2 knockout (*Cd4-Cre*, *Parp1^-/-^*, and *Parp2^f/f^*) (partial double knockout, DKO). Taking into account the involvement of both PARP1 and T cells in the inflammatory pathologies, especially in IBDs, the crossbred animal models provided us with the chance of exploring the potential alterations in the large intestines associated with PARP deficiency.

## 2 Materials and methods

### 2.1 Animal

The mice in the study were provided courtesy of Dr. José Yélamos from IMIM Hospital del Mar Medical Research Institute and Dr. Françoise Dantzer from University of Strasbourg.

Mice bearing T-cell-PARP2 knockout in a PARP1 depletion background (*cd4-Cre*, *Parp1* ^-/-^, and *Parp2*^f/f^; referred below as “DKO” mice) were generated from the crossbreeding of mice with either global PARP1 (PARP1 KO, *cd4-Cre*, *Parp1^-/-^*, and *Parp2^+/+^*) or T-cell specific PARP2 deficit (T-PARP2 KO, *cd4-Cre*, *Parp1^+/+^*, and *parp2^f/f^*). Selective PARP2 knockout in T cells was achieved by the recombination of Cre-recombinase into CD4 exon, which was also completed in the control animals (*cd4-Cre*, *Parp1^+/+^*, and *Parp2^+/+^*). The genotypes of the mice were confirmed by examining samples from the toes of mice using nucleic acid amplification (polymerase chain reaction) with designed primers and SDS-PAGE. The effect of genetic modification was confirmed in a previous study by Navarro et al. (16).

The current investigation complied with the EU Directive 2010/63/EU and the Guide for the Care and Use of Laboratory Animals published by the US National Institutes of Health (NIH Publication No. 85-23, revised 1996). The study was reviewed and approved by the Scientific Ethical Committee on Animal Experimentation (Hungary) and by the Institutional Ethics Committee of Semmelweis University (reference no. PE/EA/1652-7/2018). The animals were kept under standard conditions with 12-h-long light and dark cycles and had access to standard laboratory rodent chow and water *ad libitum* during the experimental period.

### 2.2 Tissue collection

Deep anesthesia was induced in male mice aged 14 to 18 weeks (*N* = 67) using Avertin (2,2,2- tri-bromo-ethanol, 0.375 mg/g, intraperitoneal). The circulatory system of the animals was perfused with saline solution. Subsequently, proximal and distal large intestine samples were collected from the animals and then either placed in phosphate-buffered formaldehyde or snap-frozen in liquid nitrogen and then stored at -80 °C.

### 2.3 Tissue histology evaluation

Hematoxylin–eosin-stained histological slides of large intestines of different segments were evaluated. After dehydration and fixation, the paraffinized tissue samples were sliced into histological slides of 5-µm thickness. Hematoxylin and eosin staining was accomplished after deparaffinization, and histological evaluation of intestinal specimens was conducted based on the modified assessing system published by Bita Naini and Galen Cortina (19) (**Table 1**). Taking into account the fact that the inflammation may be confined to certain location, two sections from each harvested segment (proximal and distal) of the colon were evaluated by two researchers with medical education, and three aspects were considered in the scoring criteria: crypt structural abnormality, basal lymphoplasmocytosis, and the presence of cryptitis and crypt abscess were assessed. For each aspect, the score ranges from 0 to 2 according to severity, and the sum of the three scores were assigned to every section. The sum of the scores of both section per segment was adopted for the data analysis.

**Table 1:** Histology scoring of chronic colonic inflammation.

Histologic features	Definitions	Scoring
Crypt architectural distortion	Irregularly arranged, branched crypts, irregular crypt outlines, atrophy, and crypt shortening, surface villiform changes	0—absent 1—mild 2—conspicuous
Lymphoplasmocytosis	Range: subjective increase in lymphocytes and plasma cells particularly at the base ≥ upper and middle thirds (mild) to bandlike collections separating crypts from muscularis mucosae (conspicuous)	
Cryptitis and crypt abscess	Range: Rare examples of neutrophilic infiltration of the epithelium (mild) up to frequently marked crypt involvement by neutrophils (conspicuous)	

### 2.4 ELISA measurement

The levels of inflammatory cytokines, such as TNFα and IL-17, in the supernatants from tissue homogenates were analyzed by using the ELISA method. Frozen tissues were homogenized in mixed buffer containing RIPA buffer, phosphatase, and protease inhibitors. Following sonication, the homogenates were centrifuged, and the supernatants were collected and later examined with corresponding ELISA kits (TNFα—Invitrogen Mouse TNFα ELISA Kit BMS607-3, IL17—Invitrogen Mouse IL-17 ELISA Kit BMS6001, Thermo Fisher Scientific). The cytokine levels were normalized to the total protein content of the samples, which were determined by using the bicinchoninic acid assay.

### 2.5 Immunohistochemistry

Immunohistochemistry was utilized for the analysis of tissue oxidative–nitrative stress (measuring the levels of 4-hydroxynonenal, 3-nitrotyrosine, and inducible nitric oxide synthase) and mucosal infiltration of T cell subpopulations. Heat-induced antigen retrieval was applied with a pressure cooker and Tris-EDTA retrieval solution (pH = 9), except for the HNE staining. The tissues were treated with 3% hydrogen peroxide to block the endogenous peroxidase activity, then blocked in 2.5% normal horse serum for 1 h, and subsequently incubated in antibodies overnight: anti-HNE antibody (ab46545, Abcam, Cambridge, UK; RRID: AB_722490, 1:200), anti-nitrotyrosine (AB5411, Merck Millipore, Darmstadt, Germany; RRID: AB_177459, 1:200), anti-iNOS polyclonal antibody (PA1-036, Thermo Fischer, MA, USA; RRID: AB_325773, 1:300), anti-Ly6G (551459, BD Biosciences, CA, USA, RRID: AB_394206, 1:500), anti-CD3 (IR503, Dako FLEX, Glostrup, Denmark, RRID: AB_3094578, 1:100), anti-FoxP3 rat monoclonal IgG2a kappa antibody (14-5773-82, Thermo Fisher eBioscience, RRID: AB_467576, 1:500), and anti-T-bet antibody (AB307193, Abcam, RRID: AB_2938873, 1:500). Secondary labeling was achieved with either horseradish-peroxidase-linked anti-rabbit polyclonal horse antibodies (MP-7401, Vector Laboratories, CA, USA, RRID: AB_2336529) or anti-rat polyclonal goat antibodies (MP-7404 Vector Laboratories, RRID: AB_2336531) according to the generating hosts of primary antibodies. Either gray-black-colored diamino-benzidine with nickel (Ni-DAB, SK-4100, Vector Laboratories) or brown-colored DAB was used for visualization. Hematoxylin provided counterstaining of the background in case of using DAB without nickel.

Images of the immunolabeled specimens were captured using Nikon Eclipse Ni Microscope (Nikon Instruments, Amstelveen, The Netherlands) equipped with a Nikon DS-RI2 camera and NIS-Elements BR imaging software (Nikon Instruments). For the study of tissue oxidative–nitrative stress, quantifying evaluation was achieved by measuring the non-calibrated optical density of the gray-black color in case of HNE (Ni-DAB) and brownish color in case of nitrotyrosine and iNOS in the epithelium with ImageJ Software (National Institutes of Health, Bethesda, MA, USA). In the case of T cell subpopulation infiltration, positively stained cells were analyzed with ImageJ Software, and the cell count was normalized to the surface area of the epithelium of the large intestines. For the high density of CD3-positive cells in the intestinal epithelium, the evaluation was conducted by counting positively stained cells in five high-power fields (HPF, ×400 magnification) per section instead of the whole surface area, and the mean of each section was adopted for statistical analysis.

### 2.6 Western blot analysis

#### 2.6.1 Evaluation of intracellular inflammatory signal transduction cascade members

Members of the intracellular inflammatory signal transduction were examined by Western blotting in three to eight animals per group. Tissue homogenate supernatants were heated at 100 °C for 5 min in reducing SDS sample buffer, and samples of equal protein mass were run on 4%–15% (w/v) gradient polyacrylamide gels (Bio-Rad). The separated proteins were transferred to the nitrocellulose membranes (Bio-Rad). The membranes were blocked in EveryBlot blocking buffer (Bio-Rad) for 10 min to prevent non-specific labeling and then incubated with the following monoclonal antibodies: p38 MAPK (#9212S; Massachusetts RRID: AB_330713), phospho-p38 MAPK (#4511S; RRID: AB_2139682), p44/42 ERK (#4695S; RRID: AB_390779), phospho-p44/42 ERK (#4370S; RRID: AB_2315112),NF-kB p65 (#8242S; RRID: AB_10859369), and E-cadherin (#3195; RRID: AB_2291471) (Cell Signalling, Danvers, MA, USA) in 1:1,000 dilution overnight at 4 °C. Bound antibodies were detected with enhanced chemiluminescence after incubating in horseradish peroxidase-conjugated anti-rabbit-IgG (from donkey) secondary antibody (GE Healthcare, Chicago, IL, USA, NA934V) in 1:5,000 dilution (1 h, room temperature). β-Actin was used as loading control, detected with the use of HRP-linked anti-actin antibody (ab49900, Abcam; RRID: AB_867494). Band intensity was quantified by using ImageJ software (ver. 1.53o). The films were scanned at 600 dots per inch in TIFF format. Each band was individually selected, and the peak area was acquired and quantified as arbitrary area values of each histogram thrice.

#### 2.6.2 Evaluation of PARP homologs and apoptotic and tight junction proteins

Tissue homogenate supernatants isolated from proximal and distal large intestines were heated at 70°C in reducing SDS sample buffer for denaturation of proteins. Equal amounts of proteins were loaded in NuPAGE Bis-Tris 4%–12% polyacrylamide gel (NP0323BOX, Thermo Fisher) and separated by electrophoresis. Afterward, the proteins were transferred onto nitrocellulose membranes and blocked in 10% non-fatty milk for 1 h. The membranes were incubated in primary antibodies diluted in 1% milk overnight: anti-PARP1 antibody (AB191217, Abcam, RRID: AB_2861274, 1:500), anti-PARP2 antibody (GTX01558, GeneTex, California, USA, RRID: AB_3675423, 1:500), anti-caspase-3 (9662, Cell Signalling Technology, RRID: AB_331439, 1:1,000), and anti-occludin antibody (ABT146, Sigma-Aldrich, Missouri, USA, RRID: AB_3731285, 1:1,000). Secondary labeling of bound antibodies was achieved by incubation in HRP-conjugated goat anti-rabbit secondary antibody in 1:1,000 dilution folds (#31460, Invitrogen), and Pierce™ ECL Western Blotting Substrate (#32109, Invitrogen) was utilized for visualization. Beta-actin was used as a loading control (ab49900 1:10,000 in 1% milk; Abcam, RRID: AB_867494). Band and column intensity, respectively, was evaluated by using Image Lab Software (Bio-Rad, Hercules, CA, USA).

### 2.7 Immunofluorescent staining

Colonic crypt length and proliferation rate were evaluated by immunofluorescence to detect cell nuclei and Ki-67 antigens. Tris-EDTA (pH = 9) solution was used for heat-induced antigen retrieval (pressure cooker). The specimens were blocked in 2.5% normal horse serum and then incubated with anti-Ki-67 antibody (14-5698-82, Thermo Fisher eBioscience, RRID: AB_10854564, 1:100) overnight. Secondary labeling was completed with FITC fluorophore-conjugated goat anti-rat IgG (A-11006, Thermo Fischer, RRID: AB_2534074, 1:100). DAPI containing VectaShield Antifade Mounting Medium (H-1000-10, Vector Laboratories) was utilized as counterstain, showing the cell nuclei. The enterocyte count was evaluated by counting the number of cell nuclei in one complete semi-crypt, ranging from the base of the crypt to the crypt table (due to histological process, only crypts showing complete length were selected for evaluation). A total of five to eight crypts were analyzed for each section, and the average cryptal cell count per section was analyzed. Nuclei with merged color from DAPI and FITC were considered positive for Ki-67, the ratio of which in complete crypts were also calculated (Ki-67 positive nuclei/total nuclei per crypt).

### 2.8 Data analysis

Statistical analysis was performed with GraphPad statistical software package (GraphPad Software, La Jolla, CA, USA). Kruskal–Wallis with Dunn’s *post hoc* test was adopted to evaluate the inflammatory score. Two-way ANOVA with Tukey’s *post hoc* test was utilized for the rest of the data, and variables with non-Gaussian distribution (Shapiro–Wilk test) were logarithmically transformed. A *p*-value <0.05 was considered statistically significant. Data are presented as mean ± standard deviation (SD) in the case of normal distribution variables (including transformed variables) and median with 95% confidence interval (95% CI) in the case of non-Gaussian distribution. *N* represents the number of animals per group from three to five independent experiments.

All authors have access to the study data and have reviewed and approved the final manuscript.

## 3 Results

### 3.1 PARP2 expression showed no correlation relative to PARP1 expression

**Figure 1:**
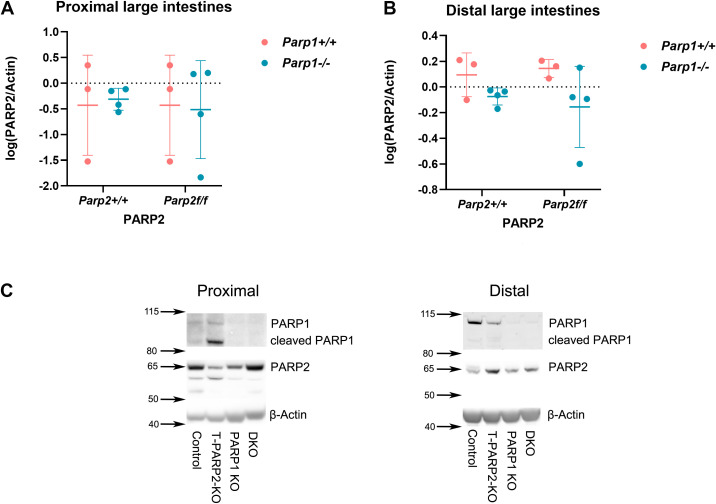
The expression of PARP1 and PAPR2 in the proximal **(A)** and distal **(B)** segments of the large intestines, respectively, was evaluated by Western blot. Relative optical density was logarithmically transformed. **(A, B)** PARP2 expression in the proximal and distal large intestines. No difference was observed among the groups of different genotypes. Dots represent individual values, and lines indicate the mean ± standard deviation (SD). **(C)** Representative Western blot images (left: proximal segment, right: distal segment) for the PARP homologs analysis. PARP1 was absent in both groups with Parp1^-/-^ genotype. β-Actin served as the loading control. The membrane was cut at 80 kDa since the visualization of PARP1 required a much longer exposure time when using a charge-coupled device, which can lead to overexposure of PARP2 and β-actin. The data were analyzed by two-way ANOVA with Tukey’s *post hoc* test. *N* = 3 to 4 animals per group.

The expression of PARP homologs in tissue homogenates was analyzed by Western blot, and the results are displayed in **Figure 1**. The expression of PARP2 was not changed in either segment of animals with the *Parp1^-/-^* genotype despite the absence of PARP1 (**Figures 1A, B**). The effect of knockout of the PARP1 gene was confirmed by densitometry, and animals with genetic manipulation showed no expression of PARP1 (**Figure 1C**).

### 3.2 DKO animals showed histological features of chronic inflammation, evaluated by inflammatory scoring

**Figure 2:**
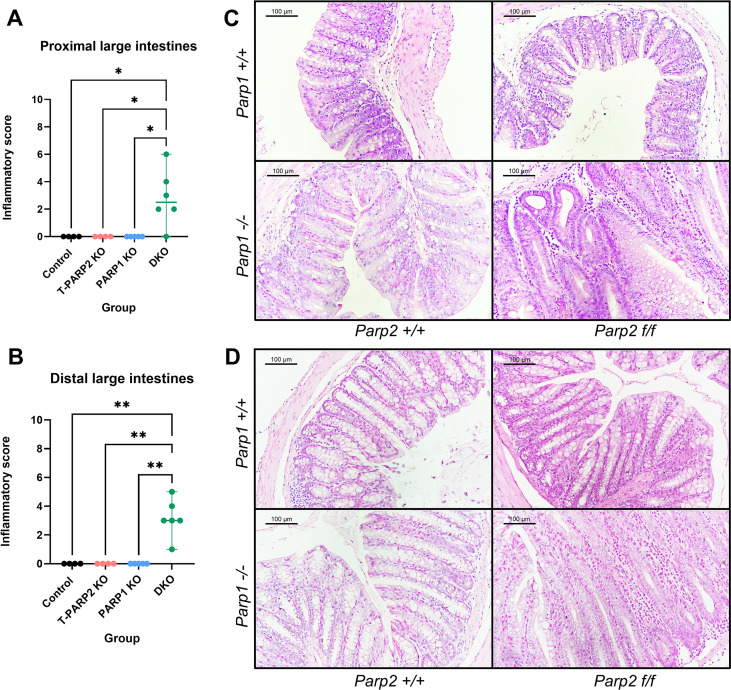
Inflammatory scores of the large intestines and representative images of H&E-stained tissue sections. **(A, B)** Inflammatory scores of sections from proximal **(A)** and distal **(B)** large intestines. The scores range from 0 to 6 for each section, and two sections were evaluated for each segment per animal. The summed results of two sections were evaluated. In both the proximal and distal large intestines, inflammation features were observed, reflected by significantly higher inflammatory scores. Dots represent individual values, and lines indicate the median with 95% confidence interval (95% CI). **(C, D)** H&E images of sections from the proximal **(C)** and distal **(D)** large intestines. The data were analyzed by using Kruskal–Wallis non-parametric test with Dunn’s *post hoc* test. The asterisk symbols represent the level of significance: *p < 0.05; **p < 0.01. *N* = 4–6 animals per group.

Microscopic examination of hematoxylin–eosin-stained tissue specimens revealed histological features resembling chronic inflammatory processes in large intestines exclusively from DKO animals, including the alteration of crypt structures, inflammatory cell infiltration, as well as the presence of cryptitis or cryptal abscess. Correspondingly, the evaluation with scoring system (according to Bita Naini and Galen Cortina) yielded higher inflammatory scores in the DKO group compared to the other groups (**Figure 2**).

Additionally, the inflammatory features appeared to be subtly different in the proximal or distal segments of the large intestines. Crypt structural abnormalities (branching crypt, irregular arrangement, etc.) and cryptitis/cryptal abscess occurred more frequently in proximal segments, while in distal segments striking thickening of epithelial mucosa was observed (elucidated in detail in the latter part).

#### 3.2.1 Levels of tissue inflammatory cytokines were altered in DKO animals

**Figure 3:**
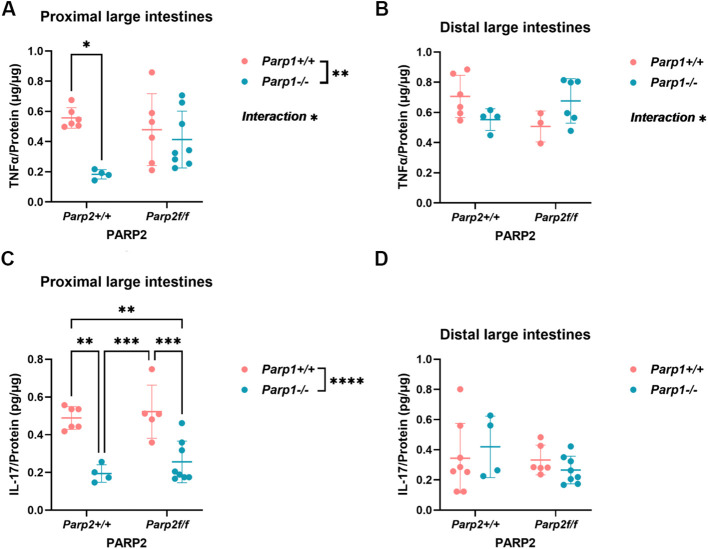
Levels of tissue inflammatory cytokines determined by using the ELISA method. **(A, B)** TNFα levels in the proximal and distal large intestines. In the proximal section, PARP1 KO animals showed a significantly lower level of TNFα compared to the control group. The PARP1 knockout has a significant effect on the TNFα levels, and the interaction between Parp1^-/-^ and Parp2^f/f^ genotypes was also significant. In the distal segments, the differences between groups did not reach the level of statistical significance, while the interaction between Parp1^-/-^ and Parp2^f/f^ genotype is also significant. **(C, D)** IL-17 levels in the proximal and distal large intestines. Animals bearing Parp1^-/-^ showed obviously lower IL-17 levels compared to animals with intact PARP1, reflecting the negative effect of PARP1 knockout on IL-17 level. In the distal segments, no alteration was observed. Dots represent individual values, and lines indicate the mean ± standard deviation (SD). The data were analyzed by two-way ANOVA test with Tukey’s *post hoc* multiple comparisons. The asterisk symbols represent the level of level of significance: *p < 0.05; **p < 0.01; ***p < 0.001; **** *p<0.0001*. *N* = 3–8 animals per group.

Inflammatory cytokine levels in the intestinal tissue were evaluated by using ELISA, and the results are shown in **Figure 3**. Significant downregulation of pro-inflammatory cytokine TNFα level was found in the proximal segments of large intestines from PARP1 KO animals compared to the control group (**Figure 3A**). In the distal segments, a similar trend could be seen despite not reaching the level of statistical significance (**Figure 3B**). A significant interaction between the *Parp1^-/-^* and *Parp2^f/f^* genotypes was identified in both segments of the intestines, suggesting that T-cell-specific PARP2 knockout reverted the suppression of the TNFα level by the absence of PARP1. For the IL-17 level, animals bearing the *Parp1^-/-^* genotypes (PARP1 KO and DKO) have a significantly lower level of IL-17 in the proximal segments compared to either group with the wild-type PARP1 genotype (PARP1 KO vs. control and T-PARP2 KO vs. DKO) (**Figure 3C**), and no difference of IL-17 was observed between groups in the distal segments (**Figure 3D**).

#### 3.2.2 Higher density of inflammatory cells was observed in the intestinal epithelium of DKO animals

**Figure 4:**
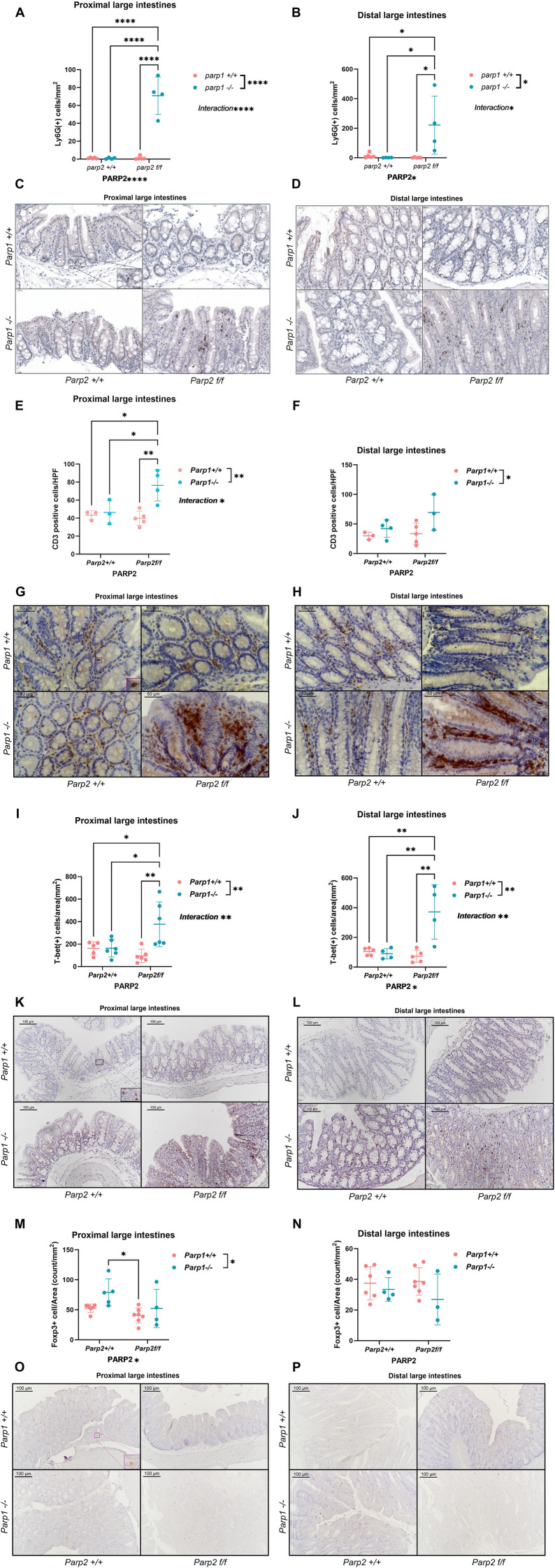
Infiltration of inflammatory cells in the epithelium of large intestines. **(A, B)** Density of Ly6G-positive cells in the epithelium of the proximal **(A)** and distal **(B)** large intestines (cell/mm^2^). The DKO animals had a higher density of Ly6G cells in the epithelium compared to the T-PARP2 KO group in both the proximal and distal segments. In both segments, Parp1^-/-^ and Parp2^f/f^ genotypes affect the density of Ly6G cells, and a positive interaction can be observed. **(C, D)**. Representative images of immunohistochemical staining against Ly6G in the proximal **(C)** and distal **(D)** segments. **(E, F)** Density of CD3-positive cells (cell per high power field (HPF), ×400 magnification) in the epithelium of the proximal and distal large intestines. In the proximal large intestines, the number of CD3-positive cells was significantly higher in the DKO group compared to the other three groups. The density of CD3-positive cells was upregulated in animals with Parp1^-/-^ genotype, and an obvious interaction between Parp1^-/-^ and Parp2^f/f^ genotypes was shown. In the distal segments, the difference did not reach the level of significance despite the positive effect of PAPR1^-/-^ remaining detectable. **(G, H)**. Representative images of immunohistochemical staining against CD3 in the proximal **(G)** and distal **(H)** segments. **(I, J)** Density of T-bet-positive cells (Th1) (cell/mm^2^) in the epithelium of the proximal and distal large intestines. In DKO animals, obvious increments in T-bet-positive cell density in both segments were observed. In both segments, two-way ANOVA revealed the significant effect of Parp1^-/-^ genotype and the interaction between Parp1^-/-^ and Parp2^f/f^ genotypes. **(K, L)** Representative images of immunohistochemical staining against T-bet in proximal **(K)** and distal **(L)** segments. **(M, N)** Density of Foxp3-positive cells in the epithelium of proximal **(M)** and distal **(N)** large intestines (cell/mm^2^). The PARP1 KO animals had a higher density of Foxp3 cells in the epithelium compared to the T-PARP2 KO group in the proximal segment, while no difference was found in the distal segment. In the proximal segments, both Parp1^-/-^ and Parp2^f/f^ genotypes affect the density of Foxp3 cells. **(O, P)** Representative image of immunohistochemical staining against Foxp3 in the proximal **(O)** and distal **(P)** segments. The brown color represents positive staining against corresponding antigens, and the blue-colored hematoxylin provided the counterstaining of the tissue. Dots represent individual values, and lines indicate the mean ± standard deviation (SD). The data were analyzed by two-way ANOVA test with Tukey’s *post hoc* test. The asterisk symbols represent the level of significance: *p < 0.05; **p < 0.01; ***p < 0.001; ****p < 0.0001. *N* = 4–8 animals per group.

The epithelial infiltration of neutrophils and T cell subpopulations was evaluated by immunohistochemistry, and the results are displayed in **Figure 4**. Neutrophils were labeled by using anti-Ly6G antibody. In both segments of the large intestines, the density of infiltrated neutrophils was significantly upregulated in the DKO animals (**Figures 4A–D**). Regarding the T cell subpopulations, the DKO animals have a higher density of CD3-positive cells in the epithelium of the proximal large intestines compared to the other three groups (**Figures 4E, G**), while in the distal segments a similar trend could be seen, although it did not reach statistical significance (**Figures 4F, H**). Nevertheless, the *Parp1^-/-^* genotype showed a consistent positive effect on the density of CD3-positive cells in both segments. The densities of T-bet positive cells (Th1) in the intestinal epithelium were markedly higher in both segments from the DKO animals compared to the other experimental groups (**Figures 4I–L**). While elevated by the *Parp1^-/-^* genotype, the density of Foxp3-positive cells (regulatory T cells) in the proximal segments was significantly downregulated by the *Parp2^f/f^* genotype. An analysis of inter-group difference only revealed a difference between the PARP1 KO and T-PARP2 KO groups (**Figures 4M, O**). In distal segments, no difference was identified (**Figures 4N, P**). The DKO animals exhibited higher oxidative–nitrative stress in their epithelium.

#### 3.2.3 DKO animals exhibited higher oxidative–nitrative stress in the epithelium

**Figure 5:**
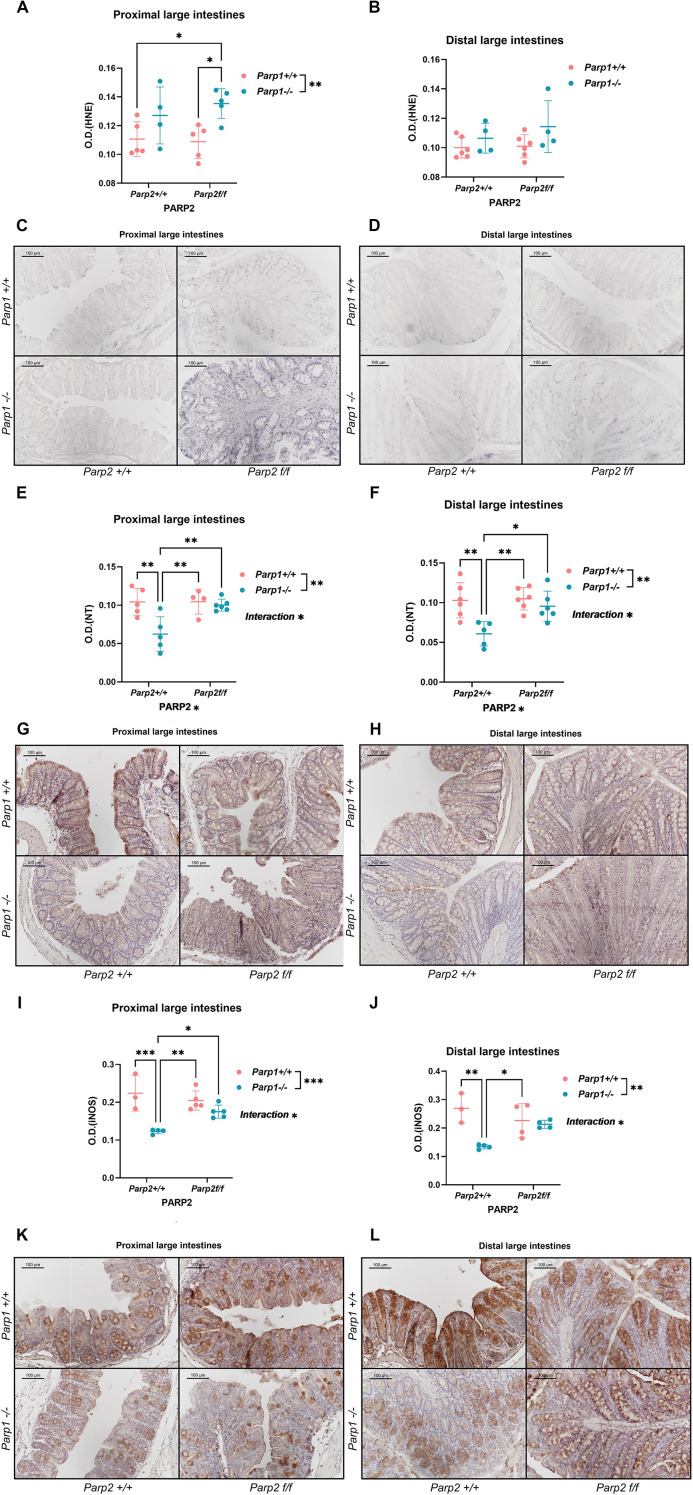
Evaluation of oxidative–nitrative markers by immunohistochemistry. **(A, B)** Epithelial level of HNE in the proximal **(A)** and distal **(B)** large intestines. In the proximal segments, the DKO animals showed significantly elevated HNE levels compared to the control and T-PARP2 KO groups. In the distal segments, no significant difference was found. Dots represent individual values, and lines indicate the mean ± standard deviation (SD). **(C, D)** Representative images of immunohistochemical staining against 4-HNE in the proximal **(C)** and distal **(D)** large intestines. The gray-black color represents positive staining. **(E, F)** Epithelial level of NT in the proximal **(E)** and distal **(F)** large intestines. In both the proximal and distal segments, the PARP1 KO animals showed lower levels of 3-NT in the epithelium compared to the other groups. Parp1^-/-^ genotype has an effect on the NT level, and the interaction between Parp1^-/-^ and Parp2^f/f^ genotypes can be seen. **(G, H)** Representative images of immunohistochemical staining against 3-NT in the proximal **(G)** and distal **(H)** large intestines. The brown color represents positive staining against corresponding antigens, and hematoxylin was used for counterstaining. **(I, J)** iNOS levels in the epithelium of proximal **(I)** and distal **(J)** large intestines. The PARP1 KO animals showed a lower level of iNOS in the proximal segments compared to the other three groups and in the distal segments compared to the control and the T-PARP2 KO groups. The negative effect of Parp1^-/-^ and the interaction between Parp1^-/-^ and Parp2^f/f^ genotypes can be seen in both segments. **(K, L)** Representative images of immunohistochemical staining against iNOS in the proximal **(K)** and distal **(L)** large intestines. The brown color represents positive staining against corresponding antigens, and hematoxylin was used for counterstaining. Dots represent individual values, and lines indicate the mean ± standard deviation (SD). The data were analyzed by two-way ANOVA, followed by Tukey’s *post hoc* test. The asterisk symbols represent the level of significance: *p < 0.05; **p < 0.01; ***p < 0.001. *N* = 3–6 animals per group.

Epithelial oxidative–nitrative stress was assessed through the evaluation of 4-hydroxynonenal (4-HNE) and 3-nitrotyrosine (3-NT) accumulation and inducible nitric oxide synthase (iNOS) level. The *Parp1^-/-^* genotype showed an upregulating effect on the 4-HNE level, while the elevation of 4-HNE level reached statistical significance only in the proximal segments of the DKO animals (**Figures 5A, C**). In the distal segment, no difference was observed (**Figures 5B, D**). A markedly decreased level of 3-NT was observed in PARP1 KO animals compared to the other three groups. However, despite also bearing the *Parp1^-/-^* genotype, the DKO animals did not show a correspondingly lowered 3-NT level; it was rather significantly higher than the PARP1 KO group, which is comparable to the two groups with the *Parp1^+/+^* genotype (**Figures 5E–H**). The level of iNOS followed the pattern of NT: in the PARP1 KO animals, it was downregulated in both segments, but in the DKO animals the level of iNOS did not differ from those of the groups with the wild-type PARP1 gene (**Figures 5I–L**).

#### 3.2.4 DKO animals showed altered intracellular inflammatory signal transduction in the proximal large intestines

**Figure 6:**
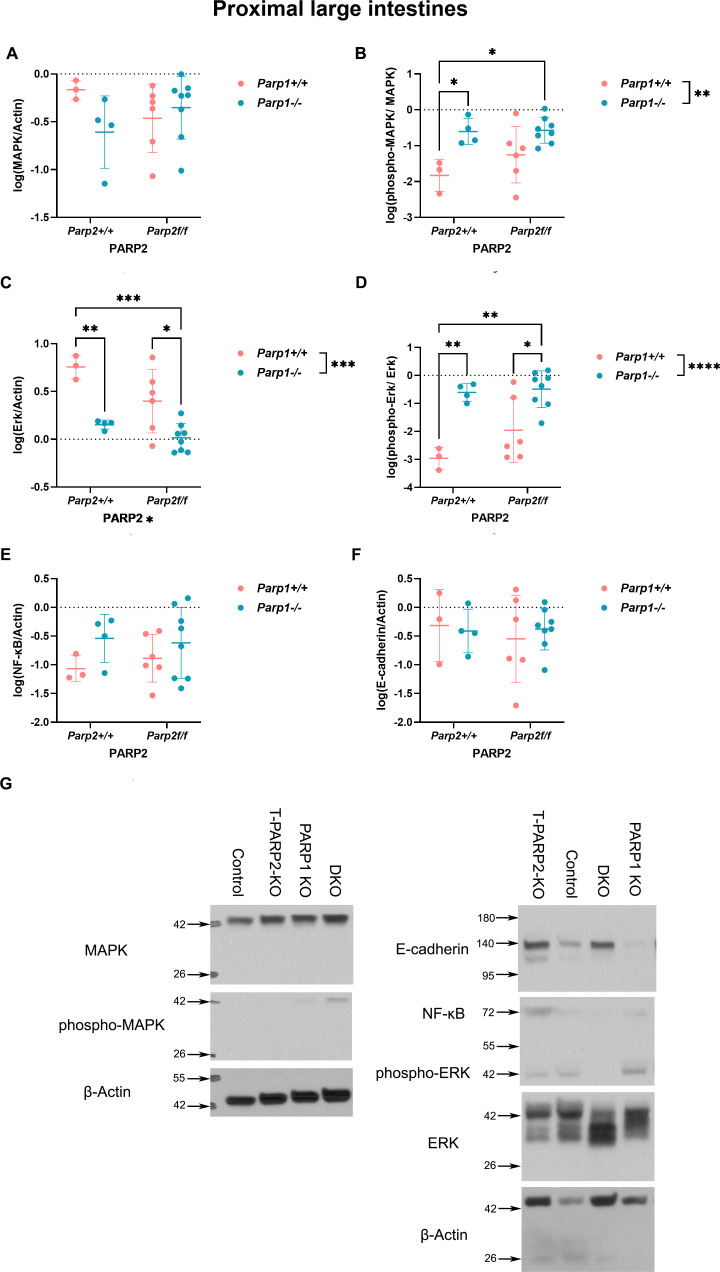
The expression and activation of members of intracellular inflammatory signal transduction proteins in the proximal large intestines were analyzed by Western blot. Relative volume intensities were logarithmically transformed. **(A, B)** Expression and phosphorylation of p38 MAPK. No difference was observed among different genotypes in the expression of MAPK, while in the groups with Parp1^-/-^ genotype (PARP1 KO and DKO) MAPK activation was significantly elevated. **(C, D)** Expression and phosphorylation of p42/44 Erk. PARP1-deficient groups (PARP1 KO and DKO) showed an obviously downregulated Erk level compared to their counterparts bearing the same PARP2 genotype, showing the significant effect of PARP1 knockout on Erk level. On the other hand, in the same groups, Erk phosphorylation was significantly upregulated compared to their relevant counterparts. **(E)** NF-κB expression. No significant alteration was observed. **(F)** E-cadherin expression. No significant difference was observed in E-cadherin level. **(G)** Representative images of Western blot membranes for protein extracts from proximal segments. β-Actin served as the loading control. Dots represent individual values, and lines indicate the mean ± standard deviation (SD). The data were analyzed by two-way ANOVA, followed by Tukey’s *post hoc* test. The asterisk symbols represent the level of significance: *p < 0.05; **p < 0.01; ***p < 0.001; ****p < 0.0001. *N* = 3–8 animals per group.

The expression and activation of intracellular inflammatory signal transduction proteins in both segments of the large intestines were studied by Western blot, and the results are displayed in **Figure 6** (proximal) and **Supplementary Figure 1** (distal). In the proximal segments, the expression of p38 MAPK did not show a difference between groups (**Figure 6A**), while its activation reflected by the ratio of phosphorylated MAPK over total MAPK was significantly higher in the groups bearing the *Parp1^-/-^* genotype (PARP1 KO and DKO) compared to the control group (**Figure 6B**). For p42/44 Erk, the total protein level in the *Parp1^-/--^*bearing groups was lower than their counterparts with matching *Parp2* genotype (control vs. PARP1 KO and T-PARP2 KO vs. DKO), showing the negative effect of the *Parp1^-/-^* genotype on the Erk levels. Besides that, the DKO animals also showed a lower Erk level in comparison to the control group (**Figure 6C**). On the other hand, the activation of Erk in groups with the *Parp1^-/-^* genotype is markedly upregulated in contrast to their counterparts, as is also the case when comparing the control and DKO groups (**Figure 6D**). The total amount of NF-κB and E-cadherin did not show any alterations (**Figures 6E, F**). The changes in MAPK and Erk, which were observed in the proximal segments, were not exhibited in the distal segments. The level of E-cadherin was shown to be influenced by the interaction between the *Parp1^-/-^* and *Parp2^f/f^* genotypes, besides the significantly downregulated level in the T-PARP2 KO group compared to the control group (**Supplementary Figure 1**).

#### 3.2.5 DKO animals showed striking hyperplasia of the cryptal enterocytes in the distal large intestines

**Figure 7:**
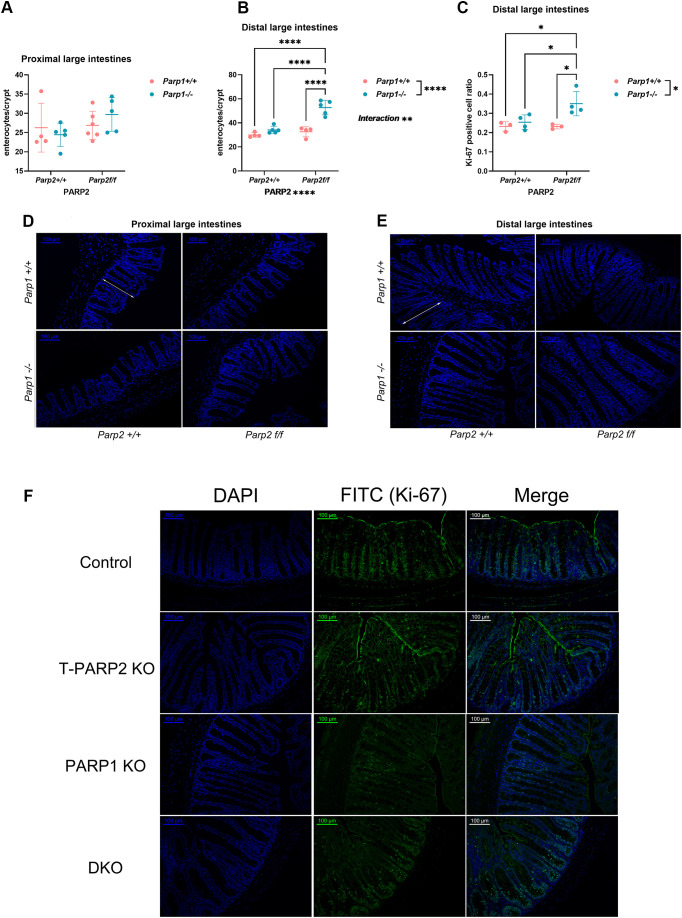
Evaluation of enterocyte count in the semi-crypts. **(A)** Enterocyte count per semi-crypt in the proximal large intestines. No difference has reached statistical significance. **(B)** Enterocyte counts per semi-crypt in the distal large intestines. The DKO animals exhibited higher enterocyte counts per semi-crypt than all other three groups. **(C)** Ki-67-positive cell ratio per crypt in the distal large intestines. The DKO animals showed a markedly higher ratio of Ki-67-positive cells per crypt in the distal segments. **(D, E)** DAPI-stained sections of the proximal **(D)** and distal **(E)** segments. The white bidirectional arrows mark examples of complete crypts included in the evaluation, the full length of which (from the base to the table of crypts) are shown. **(F)** Immunofluorescent staining images of Ki-67-positive cells in the epithelium. Ki-67 was labeled by FITC-conjugated antibody, and the cells showing merged colors of DAPI (blue) and FITC (green) were considered positive. Dots represent individual values, and lines indicate the mean ± standard deviation (SD). The data were analyzed by two-way ANOVA, followed by Tukey’s *post hoc* test. The asterisk symbols represent the level of significance: *p < 0.05; **p < 0.01; ***p < 0.001; ****p < 0.0001. *N* = 3–6 animals per group.

A histological examination of large intestinal tissue sections has revealed the presence of a thicker mucosa layer in the DKO animals, especially in the distal segments. Immunofluorescent staining was utilized to study the hyperplasia of the mucosa. The results are displayed in **Figure 7**. The average enterocyte count per semi-crypt was markedly higher in the distal segments of the large intestines from the DKO animals compared to all other groups. Both of the *Parp1^-/-^* and *Parp2^f/f^* genotypes, as well as the interaction between the two genotypes, showed an effect on the enterocyte count per semi-crypt (**Figures 7B, E**). Nevertheless, no significant difference was observed in the proximal segment (**Figures 7A, D**). Cellular Ki-67 positivity represents the ongoing cell division. The ratio of Ki-67-positive cells per crypt is also significantly higher in the distal segments of the DKO animals in comparison to other genotype groups, showing a higher renewal rate of enterocytes in the mucosa (**Figures 7C, F**).

#### 3.2.6 The level or activation of pro-apoptotic proteins is genotype and segment dependent

**Figure 8:**
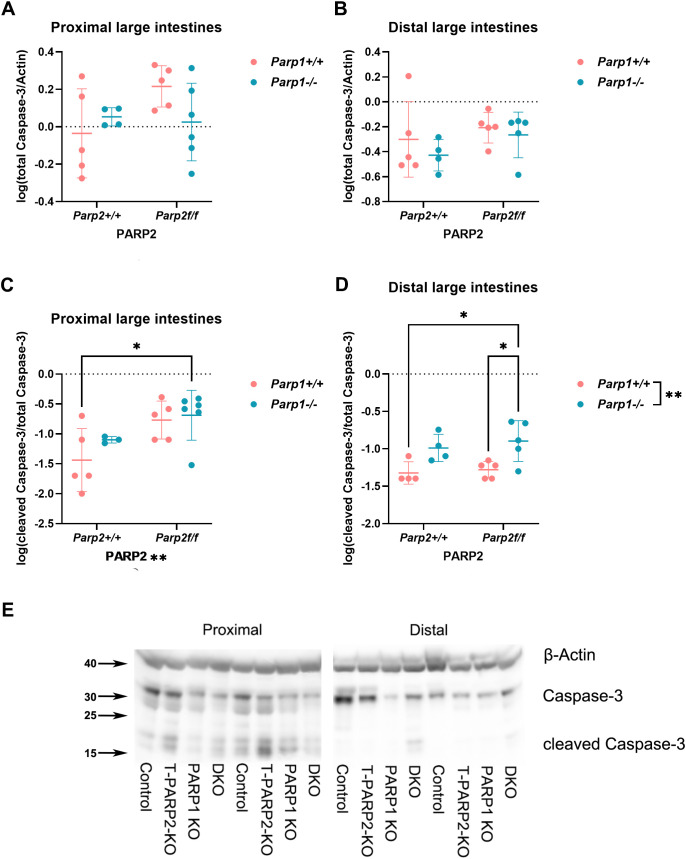
Expression and activation of caspase-3 in large intestines. **(A, B)** Expression of caspase-3 in the proximal and distal large intestines. Relative volume intensities were logarithmically transformed. No difference reached statistical significance. **(C, D)** Activation of caspase-3 in the proximal and distal large intestines, reflected by the ratio of cleaved caspase-3 over total caspase-3. In the proximal segments, the activation of caspase-3 was elevated in the DKO group compared to the control group. The Parp2^f/f^ genotype also showed significant effects on caspase-3 activation. In the distal segments, the DKO animals showed higher caspase-3 activation compared to the groups with intact PARP1 (control and T-PARP2 KO), and the Parp1^-/-^ genotype showed significant effects. The data were analyzed by two-way ANOVA with Tukey’s *post hoc* test. The asterisk symbols represent the level of significance: *p < 0.05, **p < 0.01, ***p < 0.001, ****p < 0.0001. *N* = 3–6 animals per group. **(E)** Representative images of Western blot membranes evaluating caspase-3 expression and activation. β-Actin served as the loading control. Dots represent individual values, and lines indicate the mean ± standard deviation (SD).

To study whether the changes in cell renewal was accompanied by altered apoptosis, Western blot was utilized to evaluate the expression and activation of pro-apoptotic proteins, and the results are shown in **Figure 8**. Caspase 3 expression did not show a marked difference between groups, either in the proximal or in the distal segments (**Figures 8A, B**). Caspase-3 activation in DKO, reflected by cleaved caspase-3 over the total caspase-3 level, is markedly elevated compared to the control group in the proximal segment (**Figure 8C**) and compared to the control and T-PARP2 KO groups in the distal segments (**Figure 8D**).

#### 3.2.7 Members of tight junction showed alteration mainly in the proximal segment of DKO animals

**Figure 9:**
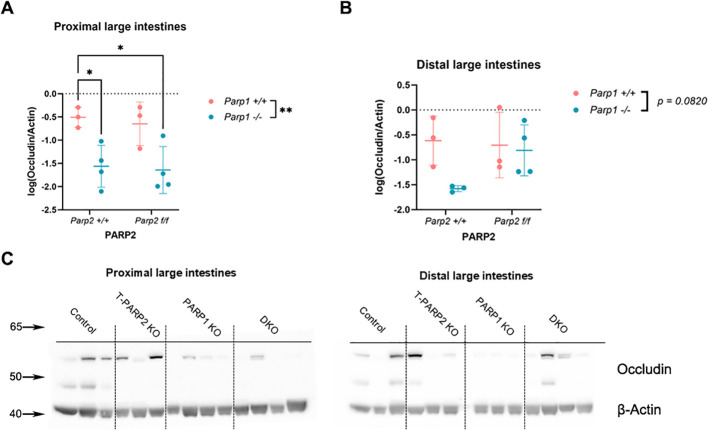
The expression of the members of tight junctions in the proximal large intestines was analyzed by Western blot. Relative volume intensities were logarithmically transformed. **(A, B)** Expression of occludin. In the proximal segment, the Parp1^-/-^genotype downregulated occludin expression, while in the distal segment the effect did not reach statistical significance. **(C)** Representative images of Western blot membranes for protein extracts from the proximal and distal segments. β-Actin served as the loading control. Dots represent individual values, and lines indicate the mean ± standard deviation (SD). The data were analyzed by two-way ANOVA, followed by Tukey’s *post hoc* test. The asterisk symbols represent the level of significance: *p < 0.05; **p < 0.01. *N* = 3 to 4 animals per group.

The expression of tight junction member occludin was studied by utilizing Western blot (**Figures 9A–C**). In proximal segments, animals with the *Parp1^-/-^* genotype showed a suppressed expression of occludin (**Figure 9A**). The downregulation of occludin did not reach statistical significance in the distal segment (**Figure 9B**).

## 4 Discussion

**Figure 10:**
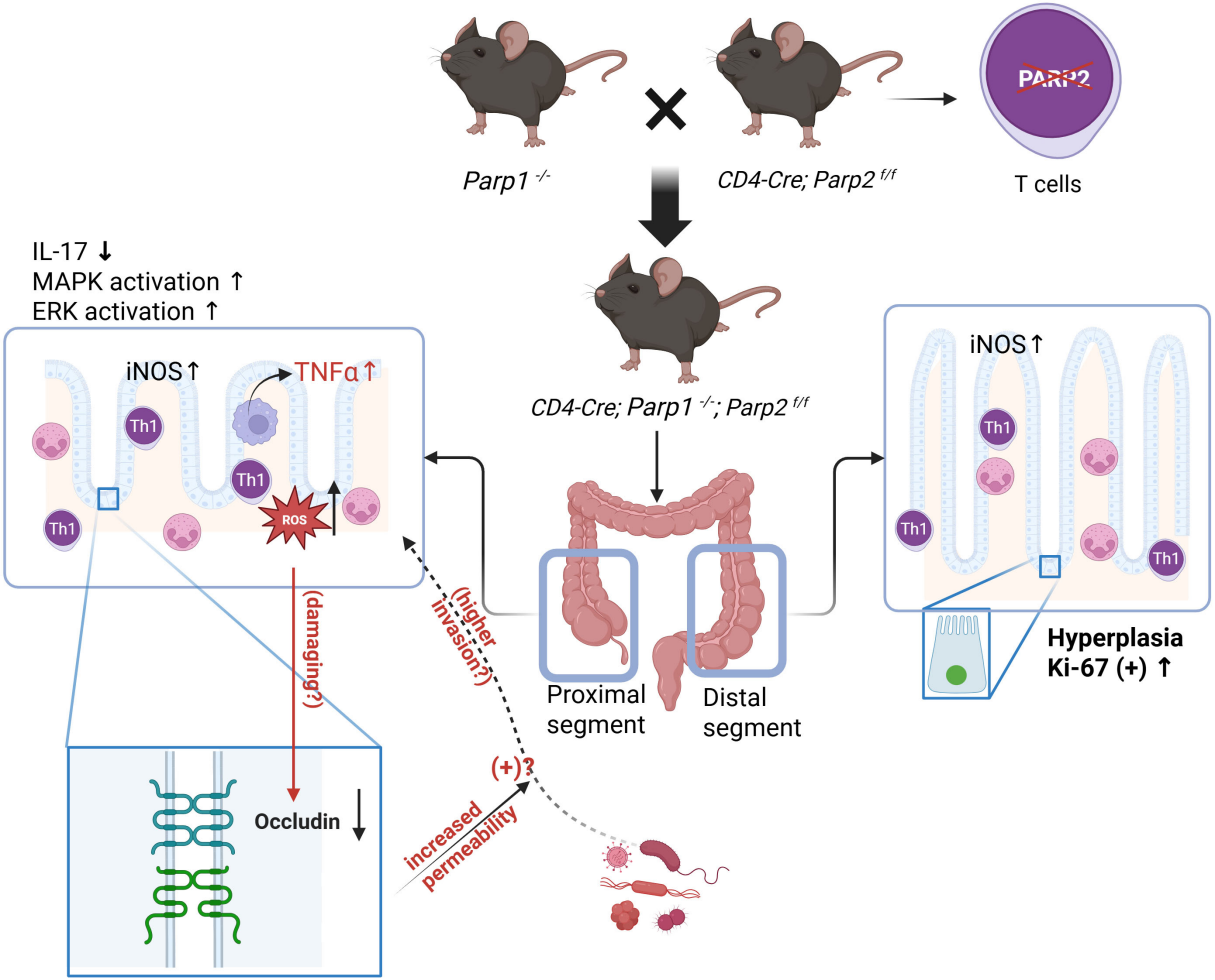
Graphic illustration summarizing the findings and possible pathophysiological process in the DKO animals. Both segments of large intestines showed increased oxidative–nitrative stress markers and infiltration of inflammatory cells like neutrophils and Th1 lymphocytes, while the proximal segment has higher TNFα and MAPK activation. IL-17 was suppressed in the proximal colon, as well as the expression of occludin, indicating an increase in permeability which may allow the increased invasion of intestinal microbial. In the distal segments, a higher epithelial turnover rate was found.

In the current study, we have demonstrated that the conditional deletion of PARP2 in T cells led to the spontaneous occurrence of mild to moderate inflammation in the large intestines of mice with a PARP1-absent background (*Cd4-Cre;Parp1^-/-^;Parp2^f/f^*) and we have summarize the results in graphic abstract displayed in **Figure 10**. The histological alterations in the mucosa, including the distortion of normal architecture, which increased the infiltration of lymphocytes, especially Th1 cell, as well as the presence of intraepithelial neutrophils and cryptal abscess have suggested the presence of active chronic inflammation. Altered levels of pro-inflammatory cytokine TNFα, elevated oxidative–nitrative stress markers, and activation of MAPK family members have validated the inflammation in corresponding segments of the intestines from various aspects. The changes in tight junction protein expression in the DKO animals reflected increased intestinal permeability, especially in the proximal segments. Additionally, we have found an increased epithelial turnover rate exclusively in the distal large intestines of the DKO animals, as reflected by the higher ratio of Ki-67-positive cells and the elevated activation of pro-apoptotic protein caspase-3. Some of the findings are of certain heterogeneity, for example, the inflammatory scores and TNFα levels, suggesting that the inflammation may not have temporally or spatially unanimous activity. Unlike chemically induced colitis, it is reasonable to see certain fluctuations in the phenotypic appearance of a chronic, spontaneously occurring intestinal inflammation, which is similar to the relapse and remission observed in patients with Crohn’s disease (20).

Beyond its critical role in the maintenance of genomic integrity, PARP1 is also involved in the regulation of both innate and adaptive immune responses. PARP1 can interact with multiple transcription factors, like NF-κB, AP-1, HMGB1, and NFAT, which regulate the expression of several inflammatory mediators or the antigenic activation of T cells. Besides that, PARP1 can alter the balance between Th1/Th2 and Th17/Treg; deletion of PARP1 can favor the transcriptional activation of Th1-related cytokines and promote the polarization of Treg (21, 22). Consequently, in multiple preclinical studies with the scope of different inflammatory pathologies, including reperfusion injuries, CNS ischemia, and experimentally induced colitis, suppressing PARP1 activity led to the amelioration of the inflammation and related injuries (4). In the studies focusing on inflammatory bowel diseases, chemicals like TNBS or DSS induced less severe colitis in the *Parp1^-/-^* animals, reflected by lower inflammatory scores, reduced expression of proinflammatory cytokines, suppressed myeloperoxidase activity, and lower oxidative/nitrative stress as well as improved intestinal permeability (22–24).

PARP2 was comparably much less studied in the frame of inflammatory bowel diseases. One study has reported that knocking down PARP2 with antisense oligonucleotides improved the spontaneous colitis in IL-10-deficit mice (12). Unlike PARP1 which is waivable for T cell, it was proven that PARP2 has a pivotal role for the maturation of T cell progenitors in the thymus. Deletion of PARP2 can lead to a higher apoptotic rate of double-positive thymocytes (14). Previously, our workgroup has found that conditional knockout of PARP2 in T cells can potentially reduce lipopolysaccharide-induced colitis (15).

Despite the protective effect against inflammatory injuries from PARP inhibition, some studies have reported contradictory results. *Parp1^-/-^* mice were proven to have earlier onset and higher disease activity in experimental autoimmune encephalomyelitis, associated with the upregulation of PARP3 mRNA levels (6). Another study has claimed that the deletion of PARP1 leads to more severe imiquimod-induced psoriasis (7). Instead of being unilaterally pro-inflammatory, PARP1 also has a rather complex role in IBD patients, which was shown by a few clinical studies. Decreased hydrogen-peroxide-induced PARP activation was found in mononuclear cells from IBD patients. Moreover, autoantibodies against F1 and F2 domains were identified in the sera of patients (8, 10, 13). One previous study from our research group revealed increased transcription levels, yet decreased translation and activity of PARP1 in the intestines from pediatric Crohn’s disease patients with active diseases (11). These preclinical and clinical data have shown that PARP1 deletion is not unanimously protective against inflammation, and certain discrepancy related to the role of PARP1 exists between the laboratory-generated animal models and the real pathology.

The immunoregulatory role of PARP1 in the intestines was reflected in our study by various data, such as the reduced level of pro-inflammatory cytokines TNFα and IL-17, lowered level of nitrotyrosine and inducible nitric oxide synthase, as well as a higher density of Foxp3^+^ cells in the proximal segments. Nevertheless, the additional depletion of PARP2 in T cells reverted the protection from the *Parp1^-/-^* genotype, leading to the upregulation of inflammatory mediators and markers in DKO animals. Such findings have suggested that PARP activity above a certain threshold in T lymphocytes has partial significance in the regulation of mucosal immunity in the large intestine. The T cells devoid of both PARP homologs were reported to have higher apoptotic rates and faulty humoral and cellular immune responses upon antigenic activation (16). In 2016, Claire BL et al. have reported that in *Rag2^-/-^* mice, where T cells rarely mature due to the absence Rag2 recombinase, dextran sulfate induced intestinal inflammation equally in both wild-type and *Parp1^-/-^* mice, as reflected by comparable histological alterations and TNFα levels, suggesting that the maturity of adaptive immunity effector is the precondition of the anti-inflammatory effect of the *Parp1^-/-^* genotype (25). Based on these discoveries, we could hypothesize that the loss of protection from the *Parp1^-/-^* genotype may be partly attributed to the disrupted immune responses of PARP1/PARP2-depleted T cells. In addition, the *Parp1^-/-^* genotype was accompanied with the downregulation of occludin, especially in the proximal segments, indicating an increase in intestinal permeability. Therefore, the faulty immune response from T cells, superimposed on increased intestinal permeability, might have predisposed the occurrence of inflammation in the large intestines of the DKO mice.

The suppressed IL-17 expression in the proximal large intestines of the DKO mice appeared to be discordant with the canonical mechanism of chronic intestinal inflammation, where excessive IL-17 expression has been considered pathogenic (3). The exact mechanism of PARP1 deletion leading to decreased IL-17 level remains elusive, while the broad suppression of immune responses from the *Parp1^-/-^* genotype may be responsible since the polarization of Th17 cells is dependent on other pro-inflammatory mediators such as IL-6 and IL-23, which are both under the transcriptional regulation of NF-κB. Yet further investigations are necessary for the clarification. Furthermore, lowering the IL-17 expression cannot be simply regarded as beneficiary for chronic colitis. Administering IL-17-neutralizing antibody to mice with DSS-induced colitis was shown to exacerbate the inflammation, while recombinant IL-17 reverted the excessive inflammatory responses (26). The clinical study from Hueber et al. investigated the efficacy of IL-17 antibody secukinumab in Crohn’s disease patients and demonstrated the striking results that neutralizing IL-17 led to increased disease activity, which excluded IL-17 antibodies from the clinical regimen of IBD therapy (27). IL-17-depleted Caco-2 cells showed altered subcellular localization of occludin, which can lead to an increase in permeability (28). Besides that, IL-17 was also reported to participate in mucosal defense against certain intestinal pathogens, which was attributed to its critical effect of promoting the expression of β-defensin in the colon (29). These findings suggested that IL-17 may possess some anti-inflammatory biological effect, and the decreased level of IL-17 in the proximal segment of the DKO animals may have even contributed to the inflammation to a certain extent.

The discrepancy between the two segments suggested the importance of studying compartmental specific responses when looking into intestinal inflammation. More severe inflammatory responses could be observed in the proximal large intestines with the evidence of larger uprising variance in the TNFα level, elevated lipid oxidative product levels, and higher activation of MAPK members. In the distal segments, epithelial hyperplasia due to increased turnover rate was found, and the suppression of IL-17 from the *Parp1^-/-^* genotype was otherwise absent. It is beyond the scope of the current study to conclude the exact mechanism behind such discordances since deeper investigations like omics studies on multiple levels would be indispensable for the clarification. Nevertheless, our findings, together with previous studies on alimentary tract compartmentalization, allow the formulation of plausible hypotheses that may guide future research. The distinction of tight junction protein expression between two segments suggested a comparably higher permeability of the proximal segments of the DKO animals, rendering these sections more vulnerable to microbial invasion. Concurrently, the disrupted T cell functions and suppressed IL-17 level might result in the overactivation of the innate immune system since certain effectors of innate immunity, such as neutrophils, are inherently devoid of PARP1, which could provide an explanation for the elevated hydroxynonenal level distinctly in the proximal segment (30). Overly produced reactive oxygen species from the immune cells could further damage the tight junctions and aggravate the rise in permeability (31). In the distal segment of the DKO animals, the occludin level was rather comparable to the control group. Therefore, the microbe of intestinal flora might have lower probability to invade through the epithelium, and less immune response will be elicited. Intestinal compartmentation shall be also considered when attempting to explain these distinctions—for example, the ratio of mucus-producing goblet cells progressively increases toward the caudal end of the intestinal tract. Mucus serves as an important barrier, and the lower production of mucus exposes the intestines to a higher risk of colitis (32, 33). The hyperplasia of the epithelium in the distal segment, which was an incidental discovery, should not be excluded from the possible explanation. The increased Ki-67-positive cell ratio and caspase-3 activation in the DKO animals indicated a higher cell turnover rate in the distal segment. Such phenotype was observed in the immune reaction against bacteria such as *Citrobacter rodentium* and *Salmonella Typhimurium* (34). Although no active infection was found or induced in our study, the mucosa constantly encounters antigen exposure from the intestinal microbiome, which might trigger immune responses that can alter the turnover rate of the intestinal epithelium. Whether this distinct alteration of the distal segment contributed to milder inflammatory reaction requires more exploration and evidence.

Overall, we have observed that the deficiency of PARP2 in T cells with global PARP1 background can lead to chronic inflammatory changes in the large intestines with aberrant features, and distinct alterations were captured in the corresponding segments of the intestines. The trigger of such inflammation remained unknown and required further investigation, while for intestinal tract facing countless antigen exposures, the potential dysregulated adaptive immune responses in the DKO animals should be studied. These findings suggest that the PARP homologs have inextricable significance for maintaining the integrity of large intestines and normal immune responses. Despite its proven protective role in colitis, overly suppressing PARP homologs in the large intestines, which is closely connected to the immune system, could result in adverse alterations in intestinal physiology.

## 5 Limitations

The current study focuses mainly on reporting the observed spontaneous inflammation in mice with T-cell-specific PARP2 deletion on a PARP1 knocked-out background and the distinct characteristics of proximal or distal segments. While the phenotypic changes associated with inflammation and segmental distinction are described in detail, the underlying mechanisms remain unresolved. As the observed alterations did not fully conform to canonical patterns, several mechanistic aspects remain open questions and can currently be addressed only at a hypothetical level. In the future, omics studies for each segment, functional study of the innate immune cells and T cell subpopulations, and dynamic intestinal permeability study could possibly bring more insight into the topic.

## Data Availability

The original contributions presented in the study are included in the article/**Supplementary Material**. Further inquiries can be directed to the corresponding author.

## Supplementary material

Supplementary Figure 1 The expression and activation of intracellular inflammatory signal transduction proteins in the distal large intestines were analyzed by Western blot. Relative volume intensities were logarithmically transformed. **(A, B)** Expression and phosphorylation of p38 MAPK. No obvious difference was observed. **(C, D)** Expression and phosphorylation of p42/44 Erk. No obvious difference was observed. **(E)** Expression of NF-κB. No significant alteration was observed. **(F)** E-cadherin expression. The T cell PARP2 knockout group showed a significantly lower E-cadherin level compared to the control group. A significant interaction between Parp1^-/-^ and Parp2^f/f^ genotypes was observed. **(G)** Representative images of Western blot membranes for protein extracts from distal segments. β-Actin served as the loading control. Dots represent individual values, and lines indicate the mean ± standard deviation (SD). The data were analyzed by two-way ANOVA, followed by Tukey’s *post hoc* test. The asterisk symbols represent the level of significance: **p* < 0.05. *N* = 3–7 animals per group.

## References

[1] Kaplan GG . The global burden of IBD: from 2015 to 2025. Nat Rev Gastroenterol Hepatol. (2015) 12:720–7. doi: 10.1038/nrgastro.2015.150. PMID: 26323879

[2] Neurath MF Finotto S Glimcher LH . The role of Th1/Th2 polarization in mucosal immunity. Nat Med. (2002) 8:567–73. doi: 10.1038/nm0602-567. PMID: 12042806

[3] Caprioli F Pallone F Monteleone G . Th17 immune response in IBD: a new pathogenic mechanism. J Crohns Colitis. (2008) 2:291–5. doi: 10.1016/j.crohns.2008.05.004. PMID: 21172226

[4] Virág L Szabó C . The therapeutic potential of poly(ADP-ribose) polymerase inhibitors. Pharmacol Rev. (2002) 54:375–429. doi: 10.1124/pr.54.3.375 12223530

[5] Laudisi F Sambucci M Pioli C . Poly (ADP-Ribose) polymerase-1 (PARP-1) as immune regulator. Endocr Metab Immune Disord Drug Targets. (2011) 11:326–33. doi: 10.2174/187153011797881184. PMID: 21476963

[6] Selvaraj V Soundarapandian MM Chechneva O Williams AJ Sidorov MK Soulika AM . PARP-1 deficiency increases the severity of disease in a mouse model of multiple sclerosis. J Biol Chem. (2009) 284:26070–84. doi: 10.1074/jbc.m109.013474. PMID: 19628872 PMC2758007

[7] Kiss B Szántó M Hegedűs C Antal D Szödényi A Márton J . Poly(ADP-ribose) polymerase-1 depletion enhances the severity of inflammation in an imiquimod-induced model of psoriasis. Exp Dermatol. (2020) 29:79–85. doi: 10.1111/exd.14061. PMID: 31755591

[8] Markowitz MM Rozen P Pero RW Tobi M Miller DG . Hydrogen peroxide induced adenosine diphosphate ribosyl transferase (ADPRT) response in patients with inflammatory bowel disease. Gut. (1988) 29:1680–6. doi: 10.1542/9781581105650-part03-ch104 PMC1434093 3146530

[9] Reumaux D Mézière C Colombel JF Duthilleul P Mueller S . Distinct production of autoantibodies to nuclear components in ulcerative colitis and in Crohn’s disease. Clin Immunol Immunopathol. (1995) 77:349–57. doi: 10.1006/clin.1995.1162. PMID: 7586746

[10] Decker P Briand JP de Murcia G Pero RW Isenberg DA Muller S . Zinc is an essential cofactor for recognition of the DNA binding domain of poly(ADP-ribose) polymerase by antibodies in autoimmune rheumatic and bowel diseases. Arthritis Rheum. (1998) 41:918–26. doi: 10.1002/1529-0131(199805)41:5<918::aid-art20>3.0.co;2-w 9588745

[11] Judit Beres N Kiss Z Muller KE Cseh A Veres-Szekely A Lippai R . Role of microRNA-223 in the regulation of poly(ADP-ribose) polymerase in pediatric patients with Crohn’s disease. Scand J Gastroenterol. (2018) 53:1066–73. doi: 10.1080/00365521.2018.1498915. PMID: 30299179

[12] Popoff I Jijon H Monia B Tavernini M Ma M McKay R . Antisense oligonucleotides to poly(ADP-ribose) polymerase-2 ameliorate colitis in interleukin-10-deficient mice. J Pharmacol Exp Ther. (2002) 303:1145–54. doi: 10.1124/jpet.102.039768. PMID: 12438538

[13] Antal D Pór Á Kovács I Dull K Póliska S Ujlaki G . PARP2 promotes inflammation in psoriasis by modulating estradiol biosynthesis in keratinocytes. J Mol Med (Berl). (2023) 101:987–99. doi: 10.1007/s00109-023-02338-z. PMID: 37351597 PMC10400701

[14] Yélamos J Monreal Y Saenz L Aguado E Schreiber V Mota R . PARP-2 deficiency affects the survival of CD4+CD8+ double-positive thymocytes. EMBO J. (2006) 25:4350–60. doi: 10.1038/sj.emboj.7601301 PMC1570435 16946705

[15] Bencsics M Bányai B Ke H Csépányi-Kömi R Sasvári P Dantzer F . PARP2 downregulation in T cells ameliorates lipopolysaccharide-induced inflammation of the large intestine. Front Immunol. (2023) 14:1135410. doi: 10.3389/fimmu.2023.1135410. PMID: 37457706 PMC10347374

[16] Navarro J Gozalbo-Lopez B Mendez AC Dantzer F Schreiber V Martinez C . PARP-1/PARP-2 double deficiency in mouse T cells results in faulty immune responses and T lymphomas. Sci Rep. (2017) 7:41962. doi: 10.1038/srep41962. PMID: 28181505 PMC5299517

[17] Moreno-Lama L Galindo-Campos MA Martínez C Comerma L Vazquez I Vernet-Tomas M . Coordinated signals from PARP-1 and PARP-2 are required to establish a proper T cell immune response to breast tumors in mice. Oncogene. (2020) 39:2835–43. doi: 10.1038/s41388-020-1175-x. PMID: 32001817

[18] Kelleher AM Setlem R Dantzer F DeMayo FJ Lydon JP Kraus WL . Deficiency of PARP-1 and PARP-2 in the mouse uterus results in decidualization failure and pregnancy loss. Proc Natl Acad Sci USA. (2021) 118(40). doi: 10.1073/pnas.2109252118. PMID: 34580230 PMC8501838

[19] Naini BV Cortina G . A histopathologic scoring system as a tool for standardized reporting of chronic (ileo)colitis and independent risk assessment for inflammatory bowel disease. Hum Pathol. (2012) 43:2187–96. doi: 10.1016/j.humpath.2012.03.008. PMID: 22703923

[20] Cho CW You MW Oh CH Lee CK Moon SK . Long-term disease course of Crohn’s disease: changes in disease location, phenotype, activities, and predictive factors. Gut Liver. (2022) 16:157–70. doi: 10.5009/gnl210118. PMID: 34456186 PMC8924800

[21] Rosado MM Bennici E Novelli F Pioli C . Beyond DNA repair, the immunological role of PARP-1 and its siblings. Immunology. (2013) 139:428–37. doi: 10.1111/imm.12099. PMID: 23489378 PMC3719060

[22] Vitali R Mancuso AB Palone F Pioli C Cesi V Negroni A . PARP1 activation induces HMGB1 secretion promoting intestinal inflammation in mice and human intestinal organoids. Int J Mol Sci. (2023) 24(8). doi: 10.3390/ijms24087096. PMID: 37108260 PMC10138503

[23] Zingarelli B O’Connor M Hake PW . Inhibitors of poly (ADP-ribose) polymerase modulate signal transduction pathways in colitis. Eur J Pharmacol. (2003) 469:183–94. doi: 10.1016/s0014-2999(03)01726-6. PMID: 12782201

[24] Zingarelli B Szabó C Salzman AL . Blockade of Poly(ADP-ribose) synthetase inhibits neutrophil recruitment, oxidant generation, and mucosal injury in murine colitis. Gastroenterology. (1999) 116:335–45. doi: 10.1016/s0016-5085(99)70130-7. PMID: 9922314

[25] Larmonier CB Shehab KW Laubitz D Jamwal DR Ghishan FK Kiela PR . Transcriptional reprogramming and resistance to colonic mucosal injury in poly(ADP-ribose) polymerase 1 (PARP1)-deficient mice. J Biol Chem. (2016) 291:8918–30. doi: 10.1074/jbc.m116.714386. PMID: 26912654 PMC4861461

[26] Ogawa A Andoh A Araki Y Bamba T Fujiyama Y . Neutralization of interleukin-17 aggravates dextran sulfate sodium-induced colitis in mice. Clin Immunol. (2004) 110:55–62. doi: 10.1016/j.clim.2003.09.013. PMID: 14962796

[27] Hueber W Sands BE Lewitzky S Vandemeulebroecke M Reinisch W Higgins PD . Secukinumab, a human anti-IL-17A monoclonal antibody, for moderate to severe Crohn’s disease: unexpected results of a randomised, double-blind placebo-controlled trial. Gut. (2012) 61:1693–700. doi: 10.1136/gutjnl-2011-301668. PMID: 22595313 PMC4902107

[28] Lee JS Tato CM Joyce-Shaikh B Gulen MF Cayatte C Chen Y . Interleukin-23-independent IL-17 production regulates intestinal epithelial permeability. Immunity. (2015) 43:727–38. doi: 10.1016/j.immuni.2015.09.003. PMID: 26431948 PMC6044435

[29] Ishigame H Kakuta S Nagai T Kadoki M Nambu A Komiyama Y . Differential roles of interleukin-17A and -17F in host defense against mucoepithelial bacterial infection and allergic responses. Immunity. (2009) 30:108–19. doi: 10.1016/j.immuni.2008.11.009. PMID: 19144317

[30] Bhatia M Kirkland JB Meckling-Gill KA . Modulation of poly(ADP-ribose) polymerase during neutrophilic and monocytic differentiation of promyelocytic (NB4) and myelocytic (HL-60) leukaemia cells. Biochem J. (1995) 308:131–7. doi: 10.1042/bj3080131. PMID: 7755555 PMC1136853

[31] Cuzzocrea S Mazzon E De Sarro A Caputi AP . Role of free radicals and poly(ADP-ribose) synthetase in intestinal tight junction permeability. Mol Med. (2000) 6:766–78. doi: 10.1007/bf03402192. PMID: 11071271 PMC1949979

[32] Johansson ME Phillipson M Petersson J Velcich A Holm L Hansson GC . The inner of the two Muc2 mucin-dependent mucus layers in colon is devoid of bacteria. Proc Natl Acad Sci USA. (2008) 105:15064–9. doi: 10.4161/gmic.1.1.10470. PMID: 18806221 PMC2567493

[33] Hansson GC . Role of mucus layers in gut infection and inflammation. Curr Opin Microbiol. (2012) 15:57–62. doi: 10.1016/j.mib.2011.11.002. PMID: 22177113 PMC3716454

[34] Sellin ME Müller AA Felmy B Dolowschiak T Diard M Tardivel A . Epithelium-intrinsic NAIP/NLRC4 inflammasome drives infected enterocyte expulsion to restrict Salmonella replication in the intestinal mucosa. Cell Host Microbe. (2014) 16:237–48. doi: 10.1016/j.chom.2014.07.001. PMID: 25121751

