# Supplementary figures and images for: Knockout of PARP2 in T cells leads to spontaneous colitis with distinct segmental characteristics in PARP1-deficient mice
Source: Front Immunol. 2026 May 19;17:1741798. doi: 10.3389/fimmu.2026.1741798 (PMC13226171; doi:10.3389/fimmu.2026.1741798)

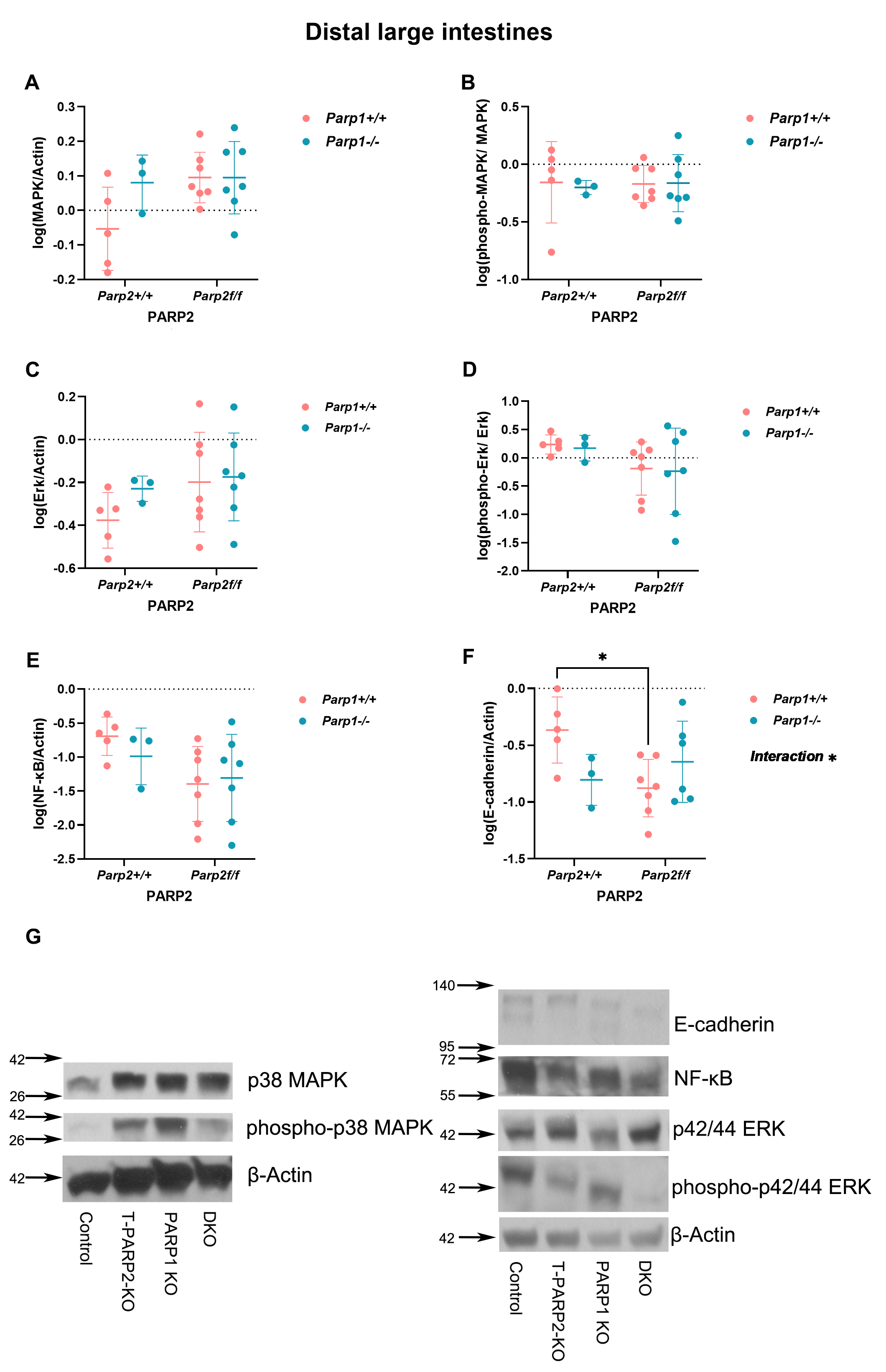

Supplement: Supplementary Figure 1 — The expression and activation of intracellular inflammatory signal transduction proteins in the distal large intestines were analyzed by Western blot. Relative volume intensities were logarithmically transformed. (A, B) Expression and phosphorylation of p38 MAPK. No obvious difference was observed. (C, D) Expression and phosphorylation of p42/44 Erk. No obvious difference was observed. (E) Expression of NF-κB. No significant alteration was observed. (F) E-cadherin expression. The T cell PARP2 knockout group showed a significantly lower E-cadherin level compared to the control group. A significant interaction between Parp1-/- and Parp2f/f genotypes was observed. (G) Representative images of Western blot membranes for protein extracts from distal segments. β-Actin served as the loading control. Dots represent individual values, and lines indicate the mean ± standard deviation (SD). The data were analyzed by two-way ANOVA, followed by Tukey’s post hoc test. The asterisk symbols represent the level of significance: *p < 0.05. N = 3–7 animals per group. [file Image1.tif]
